# Hybridization but No Evidence for Backcrossing and Introgression in a Sympatric Population of Great Reed Warblers and Clamorous Reed Warblers

**DOI:** 10.1371/journal.pone.0031667

**Published:** 2012-02-27

**Authors:** Bengt Hansson, Maja Tarka, Deborah A. Dawson, Gavin J. Horsburgh

**Affiliations:** 1 Department of Biology, Lund University, Lund, Sweden; 2 Department of Animal and Plant Sciences, University of Sheffield, Sheffield, United Kingdom; Monash University, Australia

## Abstract

Hybridization is observed frequently in birds, but often it is not known whether the hybrids are fertile and if backcrossing occurs. The breeding ranges of the great reed warbler (*Acrocephalus arundinaceus*) and the clamorous reed warbler (*A. stentoreus*) overlap in southern Kazakhstan and a previous study has documented hybridization in a sympatric population. In the present study, we first present a large set of novel microsatellite loci isolated and characterised in great reed warblers. Secondly, we evaluate whether hybridization in the sympatric breeding population has been followed by backcrossing and introgression.

We isolated 181 unique microsatellite loci in great reed warblers. Of 41 loci evaluated, 40 amplified and 30 were polymorphic. Bayesian clustering analyses based on genotype data from 23 autosomal loci recognised two well-defined genetic clusters corresponding to the two species. Individuals clustered to a very high extent to either of these clusters (admixture proportions ≥0.984) with the exception of four previously suggested *arundinaceus*–*stentoreus* hybrid birds that showed mixed ancestry (admixture proportions 0.495–0.619). Analyses of simulated hybrids and backcrossed individuals showed that the sampled birds do not correspond to first–fourth-generation backcrosses, and that fifth or higher generation backcrosses to a high extent resemble ‘pure’ birds at this set of markers.

We conclude that these novel microsatellite loci provide a useful molecular resource for *Acrocephalus* warblers. The time to reach reproductive isolation is believed to be very long in birds, approximately 5 Myrs, and with an estimated divergence time of 2 Myrs between these warblers, some backcrossing and introgression could have been expected. However, there was no evidence for backcrossing and introgression suggesting that hybrids are either infertile or their progeny inviable. Very low levels of introgression cannot be excluded, which still may be an important factor as a source of new genetic variation.

## Introduction

Hybridization and introgression can lead to the creation of novel genotypes and phenotypes and are therefore important processes in the evolution of animals and plants [1], [2]. Hybridizing species and hybrid zones provide excellent opportunities to examine evolutionary processes such as adaptation, gene flow and, ultimately, speciation [3]–[6]. Determining the degree and pattern of introgressed genetic material between recently diverged species may be particularly interesting from an evolutionary point of view, since they typically show incomplete reproductive isolation.

Studies of hybrid zones have indicated that natural hybridization is most likely to take place in intermediate habitats, which are often found at the ecological limits of the species' distributional ranges, and where both taxa are found in close proximity to each other [4]. If some of the interspecific matings lead to fertile first-generation (F_1_) hybrids, then there is a possibility that these will backcross with at least one of the parental genotypes, with introgression as a consequence. If the resulting backcrossed individuals subsequently mate with the most similar parental genotype, novel genes and gene complexes can be particularly rapidly introduced into the new genetic background [7]. In some cases, stable and long-lasting hybrid zones are formed as a consequence of spatial range overlap between two species [8]–[10]. However, another possible scenario is that one of the two species, or possibly even the new hybrid cross, becomes more successful and displaces one or both of the original taxa [11].

In birds, several well-characterised hybrid zones are known, e.g. between carrion and hooded crow (*Corvus corone* ssp.) [12], wood warblers of the genus *Denroica* [13], and Darwin's finches (*Geospiza* spp.) [14]. Avian hybridization seems to be quite commonly occurring when two related species meet and one of them is rare [15], [16]. In such situations, individuals that remain unpaired might choose heterospecific mates. Alternative hypotheses postulate that hybridizing females are attracted to heterospecific males when these are larger in size than the conspecific males, or that heterospecific song and plumage characteristics sometimes act as supernormal mate choice stimuli [17]. Hybridization might also result from general mistakes in mate recognition [17].

The great reed warbler (*Acrocephalus arundinaceus*) and the clamorous reed warbler (*A. stentoreus*) are closely related passerines in the family Sylvioidea [18]. They are similar in morphology and behaviour, and have partly overlapping breeding ranges in the Middle East and southern Central Asia [19], [20]. The great reed warbler is a long-distance migrant throughout its range, whereas clamorous reed warblers are either sedentary or perform a short-distant migration. Morphologically the two species are distinguished most easily on differences in wing characters (length and shape) [19]. In addition, males are easily distinguished by their song: the great reed warbler has a variable and high-pitched song, whereas the clamorous reed warbler has a monotone song of low frequency ([19]; B. Hansson, personal observation). In southern Central Kazakhstan, great reed warblers (subspecies *A. a. zarudnyi*) are at their south-eastern range limit, whereas clamorous reed warblers (subspecies *A. s. brunnescens*) are at their northern range limit [19], [20]. In this region, the clamorous reed warbler has expanded its range northwards during the last three decades, with increasing numbers in the newly colonized areas [21]. Currently, the breeding ranges of the two species overlap over a zone 500×1400 km wide and several sympatric breeding populations are known [20], [21]. We have previously detected that viable hybrids between these two species occur in a sympatric breeding population in Kazakhstan [21]. Birds with intermediate morphology were identified as hybrids: four of the examined individuals had intermediate wing characteristics and, based on one mitochondrial locus (the control region) and one nuclear microsatellite locus (*Ase50*), carried genetic material from both parental species [21]. It is not known, however, whether the hybrids were fertile and if backcrossing to either of the parental population occurs (cf. [22]).

In the present study, we evaluate whether ongoing hybridization in the sympatric breeding population in Kazakhstan has been followed by backcrossing and introgression of genetic material between the great reed warbler and the clamorous reed warbler. For this purpose, we isolated novel microsatellites in the great reed warbler, and evaluated a subset of them for amplification success and degree of polymorphism. We then genotyped a few already identified hybrids and a larger number of individuals characterised as either pure great reed warbler or pure clamorous reed warbler based on morphology, at a set of autosomal microsatellite loci. Bayesian clustering analyses [23] were applied aiming at distinguishing hybrids and backcrosses in the population (cf. [24], [25]). Furthermore, we created simulated hybrid and backcross genotypes to understand the expected genetic signature of hybridization and introgression. We conclude that our novel microsatellites provide a useful genetic resource for these warblers, and that there is no evidence for backcrossing and introgression in the study population.

## Materials and Methods

### Study species, field work and DNA extraction

Great reed warblers breed in lakes and marshes throughout the Eurasia and migrate to spend the winter in Africa south of the Sahara [19]. Currently, two subspecies are recognised, *A. a. arundinaceus* in the western part of the range and *A. a. zarudnyi* in the east. The species is facultatively socially polygynous [19], [26]. Habitat requirements of the closely related clamorous reed warbler are similar to those of the great reed warbler, although this species is also found in less vegetated wetlands. Four subspecies are distinguished of which *A. s. brunnescens* occurs in southern Central Asia. This subspecies performs a short-distance migration mainly to the Indian sub-continent, whereas other subspecies are sedentary [19], [27]. Great reed warblers and clamorous reed warblers differ in length and structure of the wings and there are also measurable differences in bill-head size and tail length [19], [21]. Some plumage differences occur. For example, the great reed warbler is greyish brown on the mantle, whereas the clamorous reed warbler is buffish brown.

We studied great reed warblers (*A. a. zarudnyi*) and clamorous reed warblers (*A. s. brunnescens*) at Stone Lake (42°51′N, 70°58′E) and Kremenevskyi pond (42°35′N, 70°39′E), located 39 km apart in southern Central Kazakhstan, where they co-occur with a total population size of approximately 500 and 40 territorial males, respectively [21]. The birds arrive to this region from mid-April to early May, clamorous reed warblers about a week ahead of the first great reed warblers [28]. Both species prefer to breed in reed beds and their territories are found side by side; in dense breeding populations the territories of con- and heterospecific males are often less than 10 m wide (B. Hansson, pers. obs.). During the period 12–19 May 2001, we captured, ringed and measured as many birds as possible; initially at Kremenevskyi pond and then, to increase the number of examined birds, also at Stone Lake. Most individuals were captured in stationary mist nets in the centre of the breeding localities. A few birds (six great reed warblers and one hybrid) were captured in mist nets within their territories using song play back. We examined 30 great reed warblers, 215 clamorous reed warblers and four putative hybrids.

At examination, a small amount of blood (<25 µl) was taken from the brachial vein of 56 birds (29 great reed warblers, 23 clamorous reed warblers and 4 putative hybrids). Blood samples were stored in SET buffer (0.15 m NaCl, 0.05 m Tris, 0.001 m EDTA). Genomic DNA was extracted using a standard phenol/chloroform protocol [29]. In a previous study [21], hybrids had been verified by genotyping at a Z-linked microsatellite marker (*Ase50* [30]), the species identity of the mother had been verified by sequencing a part of a the mitochondrial control region (using the primers BCML4 and 12SH1 [31]), and the sex of the birds had been identified by amplifying a Z- and W-linked locus (*CHD1Z/W* using the primers 2550F and 2718R [32]).

### Microsatellite isolation

A microsatellite-enriched library was constructed from a single female ‘pure’ great reed warbler sampled at Lake Kvismaren, Sweden (see e.g. Hasselquist 1998 [26]). The library was constructed at NERC Biomolecular Analysis Facility at the University of Sheffield. The method of Armour *et al.* [33] was used and the *Mbo*I fragments were enriched separately for the following di- and tetra-nucleotide microsatellite motifs: (GT)_n_, (CT)_n_, (GTAA)_n_, (CTAA)_n_, (TTTC)_n_ and (GATA)_n_ and their complements, which had been denatured and bound to magnetic beads following Glenn & Schable [34]. Transformant colonies were not screened for the presence of a repeat region but were directly sequenced by the NERC Biomolecular Analysis Facility at the University of Edinburgh. Clones were sequenced in the forward and reverse orientation and a consensus sequence created.

A total of 181 unique great reed warbler microsatellite sequences were isolated (EMBL accession numbers: FM878097–FM878277; Table S1). The location of these loci on the zebra finch (*Taeniopygia guttata*) genome assembly (tgu3.2.4, build 1.1; [35]), based on BLAST analyses (E-value<1E-10), is provided in Table S1 and illustrated in Figure S1. PCR primers were successfully designed for most of the 181 loci (Table S2) using PRIMER3 [36], and 41 loci were tested for amplification and polymorphism in four unrelated great reed warbler individuals from Lake Kvismaren (Table S2). All loci were PCR-amplified using Qiagen Multiplex PCR kit (Qiagen, Ltd.). The following PCR conditions were used: pre-heating for 95°C for 15 min, then 35 cycles at 94°C for 30 s, annealing temperature (*T_a_*) for 90 s, 72°C for 60 sec, followed by 60°C for 30 min and an ambient hold temperature (locus specific *T_a_* is given in Table S2). PCR products were separated using an ABI Prism 3730 capillary Sequencer and analyzed with GeneMapper 4.0 (Applied Biosystems).

We genotyped 28 great reed warblers, 15 clamorous reed warblers and the 4 previously detected hybrids in the Kazakhstan population, using 19 of the newly isolated loci and four other published microsatellite loci known to be polymorphic in great reed warblers (Table S3). The amplification conditions were as described above and *T_a_* is given in Table S2.

### Population genetic analyses

Each locus was tested for deviation from Hardy-Weinberg equilibrium using the software Fstat version 2.9.3 [37], and estimates of null allele frequencies (Oosterhout's estimate) were conducted using Micro-Checker [38]. Fstat was further used to calculate several basic measures of genetic diversity, including expected heterozygosity (H_EXP_), number of alleles and Wright's inbreeding coefficient (F_IS_).

To detect potential hybrids and backcrosses, we performed ‘admixture models’ in Structure (ver. 2.3.3; [23]). Allele frequencies were allowed to be ‘correlated’ in the models. We started each run with a ‘burn-in’ period of 50,000 replicates, followed by a sampling period of 50,000 replicates. In this study we were particularly interested in the results from the runs with two clusters (*K* = 2), corresponding to the two species. However, we also tested *K* from 1 to 5, and as expected these analyses confirmed that *K* = 2 is the most likely *K* in our data (see Results). The admixture proportion (±90% credible intervals) of each individual to the genetic clusters reflects the degree of genetic similarity to the two species, and hybrids are expected to show intermediate values and genetically pure individuals values close to one (cf. [24], [25]).

To understand the power to detect hybrids and backcrosses with our set of markers and degree of divergence of the two species (cf. [25]), we generated simulated genotypes of hybrids and backcrosses with the program Hybrid-lab [39] using the genotypes of the 28 individual great reed warblers and the 15 clamorous reed warblers as initial inputs. We generated 100 genotypes of each of the following crosses: first-generation hybrid (F1), and first to fourth generation backcross to both paternal species. We then evaluated the admixture proportions (±90% credible intervals) of these artificial crosses with Structure using similar admixture models but this time we used the ‘population flag’ option, which allows clusters to be based on allele frequencies from pre-specified reference populations; in our case great reed warblers and clamorous reed warblers, hence *K* = 2 (cf. [24], [40], [41]).

## Results

### Genetic variation at the loci

Of the 41 novel microsatellite loci tested, 40 (98%) amplified and 30 (73%) were polymorphic in four unrelated great reed warbler individuals from Sweden (Table S2).

In the 28 great reed warblers from Kazakhstan categorised as pure species based on morphology, the number of alleles per locus ranged from 2 to 26 with a mean of 7.96 at a set of 23 autosomal loci (19 of the newly isolated loci and four other published microsatellite loci known to be polymorphic in great reed warblers; Table S3). Expected heterozygosity ranged from 0.14 to 0.97, and F_IS_ was low to moderate for all loci (Table S3). Significant deviation from Hardy-Weinberg equilibrium due to homozygous excess was found for locus *Aar39* (F_IS_ = 0.25; *P* = 0.016), and the Micro-Checker analyses suggested the presence of null alleles at this locus at a frequency 12% (Table S3).

All 23 primer pairs cross-amplified in the clamorous reed warbler and the number of alleles ranged from one to 12 with a mean of 5.57, whereas the expected heterozygosity ranged from 0 to 0.97 with a mean of 0.55 (Table S3). Significant deviation from Hardy-Weinberg equilibrium due to homozygous excess was found at two loci, *Aar33* and *Aar57*, in clamorous reed warbler (Table S3).

### Bayesian clustering analyses

The Structure analyses detected strong genetic differentiation between the two species. The estimated likelihood probability of the data, LnP(D), for *K* = 1–5 was as follows: −3494.4, −3064.5, −3509.7, −3175.8, and −3256.8. Thus, *K* = 2 was the most likely number of genetic clusters in the group of individuals genotyped.

**Figure 1:**
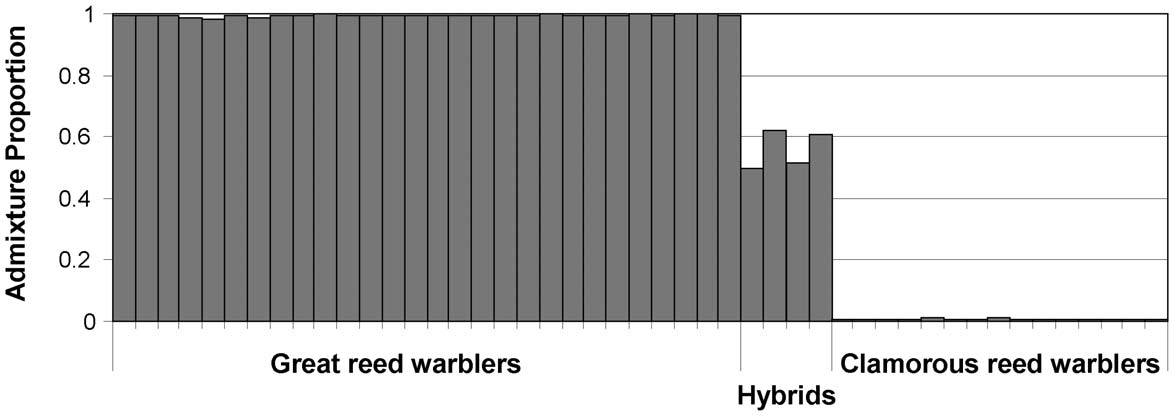
Admixture proportions of great reed warblers, clamorous reed warblers and hybrids in a sympatric breeding population in Kazakhstan to two genetic clusters generated from admixture models using the Bayesian genetic clustering technique implemented in Structure.

The individual admixture proportions indicate that there are no backcrossed individuals present in the sample and there is no evidence for introgression of genetic material between the species (Figure 1). The 28 great reed warblers had admixture proportions ≥0.984, and for the clamorous reed warblers the corresponding values were ≥0.991 (Figure 1). In contrast, the four individuals that were categorised as hybrids based on intermediate morphology (and the genetic signature at one mitochondrial and a single microsatellite locus [21]) had admixture proportions of between 0.495–0.619 to the cluster corresponding to the great reed warbler genotypes (Figure 1).

**Figure 2:**
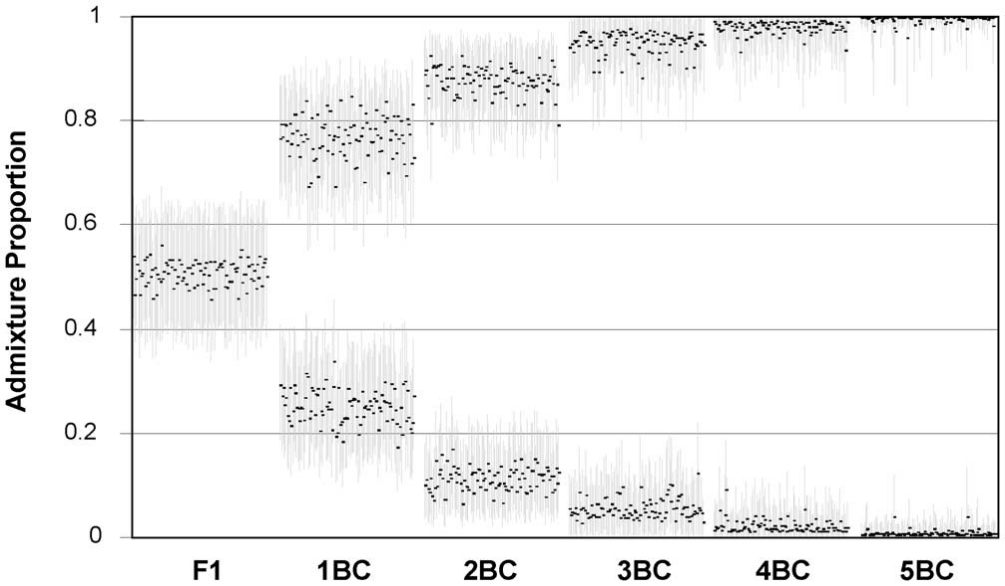
Admixture proportions and 90% credible regions to two genetic clusters of simulated hybrids (F1) and first–fifth generation backcrosses (1-5BC; 100 individuals in each category) between great reed warblers and clamorous reed warblers. The higher the admixture proportion, the higher the similarity to the genetic cluster corresponding to a ‘pure’ great reed warbler genotype.

The analyses of the artificial hybrid and backcross genotypes (Figure 2) suggest that the four hybrids are similar to genotypes expected for F1 hybrids and less likely to be first or higher generation backcrosses, and furthermore that the individuals categorised as pure species based on morphology are genetically as pure or purer than fifth generation backcrosses (Figure 1; Figure 2).

## Discussion

In the present study, we have presented a large set of novel microsatellite loci isolated from and characterised in the great reed warbler. In total, 181 loci were isolated and, among 41 tested for amplification, 73% were polymorphic in a small set of great reed warbler individuals. Moreover, we used a set of 23 autosomal microsatellites to evaluate whether ongoing hybridization in a sympatric breeding population in Kazakhstan has been followed by backcrossing and led to significant introgression of genetic material between the species. The clamorous reed warbler has expanded its range northwards in Kazakhstan during the last three decades and sympatric breeding populations have been recorded since 1981 [21]. Within a sympatric locality, the two species show no or little habitat separation and they take up neighbouring territories within their preferred reed habitat (B. Hansson, pers. obs.). Thus, there should have been plenty of opportunities for backcrossing and introgression in those areas where the two species overlap.

We used Bayesian clustering analyses in the program Structure to recognise two genetic clusters corresponding to the two species, and all individuals either clustered to a very high extent to either of the species cluster (i.e. ‘pure’ species) or showed a mixed ancestry (i.e. hybrids). There was neither any evidence for backcrossed individuals nor introgressed genetic material in the population, suggesting that the hybrids are either infertile or their progeny inviable. We cannot of course exclude very low levels of backcrossing and old introgression events in the study populations (cf. [25]), which still may be an important factor as a source of new genetic and phenotypic variability [3], [42]. Nevertheless, there seem to be little potential adaptive significance of introgression in these *Acrocephalus* warblers.

In vertebrates, hybridization is particularly common in fish, where several hundred interspecific and intergeneric crosses have been reported, and in birds with roughly 10% of all species known to have bred in the wild with another species (e.g. [4], [16], [43]). As mentioned above, the breeding ranges of great reed warblers and clamorous reed warblers overlap in southern Kazakhstan and a previous study has documented the occurrence of hybrids in a sympatric population [21]. Occasionally, heterospecific matings and/or viable hybrids have been documented also between other *Acrocephalus* species [19], [44], for example, between reed warbler and marsh warbler, *A. scirpaceus* and *A. palustris* [45], and between reed warbler and great reed warbler [46], [47].

Previous work on plants [48], [49] and animals [16] has suggested that directional hybridisation usually occurs between the females of the rare species and the males of a common species, but not vice versa. Consequently, under such a condition, the rare species is usually the maternal parent of the hybrids. This is not the case in the present study system, where females of both the rarer species (great reed warbler) and the more common species (clamorous reed warbler) are known to engage in hybrid matings [21]. Interestingly, three of the four hybrids in the data set were previously found to carry clamorous reed warbler mitochondria, and, hence, had clamorous reed warbler mothers [21]. At the breeding locality, there were about eight times more clamorous reed warblers than there were great reed warblers, but despite this both species were common. There was no indication of any difference in sex-ratio between species (B. Hansson et al., unpublished). Therefore, the data from this population neither support the hypothesis suggesting that females of the rarer sex should be engaged in hybrid matings, nor the hypothesis proposing that hybridization happens when either species is rare [15], [16], [21].

The *cytochrome b* sequence divergence between great reed warbler (GenBank sequence accession record: AJ004784) and clamorous reed warblers (AJ004788) is approximately 4% which may correspond to a divergence time of approximately 2 Myrs [50]. The time to reach complete reproductive isolation, with hybrids of both sexes being fully infertile, is very long in birds; it was recently estimated to be approximately 5 Myrs based on data from several taxa [43]. Although this estimate should be taken only as a rough indication in each particular case, with an estimated divergence time of 2 Myrs between great and clamorous reed warblers backcrossing and introgression would not have been unlikely. In some other similar study systems a low degree of introgression has in fact been detected. For instance, in icterine and melodious warblers (*Hippolais icterina* and *H. polyglotta*), and between collared and pied flycatchers (*Ficedula albicollis* and *F. hypoleuca*) introgression occurs at low frequencies [10], [51].

We conclude that our novel microsatellite markers provide a useful molecular genetic resource for this group of birds, and that there is no evidence for backcrossing and introgression in the study population, which in turn suggests that hybrids between these two warbler species are either infertile or their progeny inviable. We cannot however exclude very low levels of introgression, which could potentially be an important factor as a source of new genetic variation in the species.

## Supporting Information

Table S1 **Summary of BLAST analyses of 181 great reed warbler (** ***Acrocephalus arundinaceus*** **) microsatellite sequences and four other avian microsatellite loci used in the present study against the zebra finch genome assembly (tgu3.2.4, build 1.1).** BLAST hit statistics and chromosomal locations in the zebra finch genome are included.
(PDF)

Table S2 **Characteristics of 181 great reed warbler (** ***Acrocephalus arundinaceus*** **) microsatellite loci isolated and characterised in this study.** Primer sequences, expected and observed product size, melting (*T*_m_) and annealing (*T*_a_) temperatures, 5′-fluoro-label, and amplification success in four test individuals are shown.
(PDF)

Table S3 **Number of alleles, range of fragment length (bp), expected and observed heterozygosity, Wright's F_IS_, and two-tailed** ***P*** **-value for deviations of Hardy-Weinberg equilibrium (** ***P*** **_HW_; shown when** ***P*** **<0.1) in great and clamorous reed warblers.** Also given are null allele frequencies in the great reed warbler.
(PDF)

Figure S1 **Chromosomal locations of great reed warbler (** ***Acrocephalus arundinaceus*** **) microsatellite loci and four other avian microsatellite loci included in the present study (Table S1) on the zebra finch (** ***Taeniopygia guttata*** **) genome.**
(PDF)
