# Supplementary material for: Hybridization but No Evidence for Backcrossing and Introgression in a Sympatric Population of Great Reed Warblers and Clamorous Reed Warblers
Source: PLoS One. 2012 Feb 27;7(2):e31667. doi: 10.1371/journal.pone.0031667 (PMC3288047; doi:10.1371/journal.pone.0031667)
Supplement: Table S1 — Summary of BLAST analyses of 181 great reed warbler ( Acrocephalus arundinaceus ) microsatellite sequences and four other avian microsatellite loci used in the present study against the zebra finch genome assembly (tgu3.2.4, build 1.1). BLAST hit statistics and chromosomal locations in the zebra finch genome are included. (PDF) [file pone.0031667.s001.pdf]

Table S1 Summary of BLAST analyses of 181 great reed warbler (*Acrocephalus arundinaceus*) microsatellite sequences and four other avian microsatellite loci used in the present study against the zebra finch genome assembly (tgu3.2.4, build 1.1). BLAST hit statistics and chromosomal locations in the zebra finch genome are included. **Summary methods:** http://www.ensembl.org/Multi/blastview; Search tool: BLASTN; Search sensitivity: Distant homologies; Single hit criteria: E-value <1E-10; E-value 1E-10 lower, or score 100 higher, than second hit; double hits (not matching the single hit criteria) are given (grey shading) when (i) there are hits to both a chromosome and chromosome Unknown, and (ii) when there are two hits on the same chromosome.

Clone No.	Clone ID	Accession number	Locus name	No. of hits	No. of chrs with hit	TguChr	Start	E-value	Score	Length
1	25_A01	FM878097.1	Aar9	2	1	1	78662579	1.70E-67	264	377
1	25_A01	FM878097.1	Aar9			1	78672118	6.40E-67	262	377
2	25_A02	FM878098.1	Aar10	1	1	2	154345106	9.40E-22	77	79
3	25_A04	FM878099.1	Aar11	1	1	3	23940825	6.30E-92	230	325
4	25_A05	FM878100.1	Aar12	1	1	1	23935630	6.90E-24	72	85
5	25_A06	FM878101.1	Aar13	1	1	5	45747352	6.40E-28	123	153
6	25_A07	FM878102.1	Aar14	1	1	4	61150538	2.00E-93	205	260
7	25_B03	FM878103.1	Aar15	1	1	4	46186384	1.30E-54	144	220
8	25_B06	FM878104.1	Aar16							
9	25_B08	FM878105.1	Aar17							
10	25_B11	FM878106.1	Aar18	1	1	7	22168918	2.20E-93	279	374
11	25_B12	FM878107.1	Aar19	1	1	11	21086707	6.70E-59	160	224
12	25_C01	FM878108.1	Aar20							
13	25_C02	FM878109.1	Aar21	1	1	1	3265906	1.50E-30	84	215
14	25_C05	FM878110.1	Aar22	1	1	1A	33601611	7.80E-53	175	220
15	25_C07	FM878111.1	Aar23							
16	25_C09	FM878112.1	Aar24	1	1	2	57618520	4.20E-77	154	206
17	25_C12	FM878113.1	Aar25							
18	25_D02	FM878114.1	Aar26	1	1	Z	17576687	3.00E-90	241	378
19	25_D03	FM878115.1	Aar27	1	1	6	16935712	2.40E-77	215	272
20	25_D04	FM878116.1	Aar28							
21	25_D09	FM878117.1	Aar29	1	1	1A	13703457	6.10E-81	249	374
22	25_D11	FM878118.1	Aar30	1	1	4	54326157	5.80E-43	177	275
23	25_E04	FM878119.1	Aar31	1	1	5	54112319	2.90E-109	233	267
24	25_E07	FM878120.1	Aar32							
25	25_E08	FM878121.1	Aar33	1	1	2	120725528	1.00E-30	109	111
26	25_E09	FM878122.1	Aar34	1	1	4	41673588	2.80E-37	156	207
27	25_E10	FM878123.1	Aar35							
28	25_F01	FM878124.1	Aar36	1	1	1	38287592	4.70E-80	234	312
29	25_F02	FM878125.1	Aar37	1	1	3	73217086	7.40E-63	224	317
30	25_F03	FM878126.1	Aar38	1	1	3	17786608	9.00E-59	222	246
31	25_F05b	FM878127.1	Aar39	1	1	3	41866704	1.30E-97	326	334
32	25_F08	FM878128.1	Aar40	1	1	19	9526604	8.60E-17	82	106
33	25_F10	FM878129.1	Aar41	1	1	4	68635663	4.20E-39	158	240
34	25_F12	FM878130.1	Aar42	1	1	3	61775447	2.30E-93	355	497
35	25_G01	FM878131.1	Aar43	1	1	2	87604336	1.50E-54	195	362
36	25_G02	FM878132.1	Aar44	1	1	Z	35619640	3.90E-55	190	282
37	25_G04	FM878133.1	Aar45	1	1	1	109851224	7.00E-141	417	504
38	25_G06	FM878134.1	Aar46	1	1	3	63707888	3.90E-50	181	276
39	25_G07	FM878135.1	Aar47	1	1	12	16121404	8.30E-111	239	328
40	25_G08	FM878136.1	Aar48							
41	25_G11	FM878137.1	Aar49	1	1	Z	40039853	1.30E-45	194	266
42	25_H04	FM878138.1	Aar50	1	1	1	67643030	7.60E-49	169	231
43	25_H05	FM878139.1	Aar51	1	1	Z	9048311	1.00E-46	144	193
44	25_H07b	FM878140.1	Aar52							
45	25_H09	FM878141.1	Aar53							
46	25_H11	FM878142.1	Aar54	1	1	3	50491696	2.40E-43	171	207
47	26_A01	FM878143.1	Aar55							
48	26_A04	FM878144.1	Aar56	1	1	4A	14432228	5.20E-49	143	191
49	26_A06	FM878145.1	Aar57	1	1	1	103980305	7.00E-76	173	236
50	26_A07	FM878146.1	Aar58	1	1	6_random	869614	2.90E-82	303	357
51	26_A08	FM878147.1	Aar59	1	1	Z	60274926	1.50E-83	243	330
52	26_A10	FM878148.1	Aar60	1	1	12	2066012	6.30E-70	263	340
53	26_A12	FM878149.1	Aar61							
54	26_B06	FM878150.1	Aar62	1	1	2	77041503	5.30E-31	108	134
55	26_B10	FM878151.1	Aar63	1	1	2	76894740	9.00E-40	105	161

56	26_B11	FM878152.1	Aar64	1	1	1	29414305	1.50E-30	103	140
57	26_C01	FM878153.1	Aar65	1	1	12	16818782	1.50E-133	479	593
58	26_C03	FM878154.1	Aar66	1	1	2	31094360	4.60E-35	151	209
59	26_C07	FM878155.1	Aar67	1	1	5	59099169	5.90E-17	82	106
60	26_C08	FM878156.1	Aar68	2	2	1A	7319474	2.70E-61	215	287
60	26_C08	FM878156.1	Aar68			Un	14676603	1.30E-59	219	287
61	26_C09	FM878157.1	Aar69	1	1	7	24293390	1.20E-79	232	300
62	26_C11	FM878158.1	Aar70	2	2	2	154055159	2.90E-124	246	290
62	26_C11	FM878158.1	Aar70			Un	40355676	9.50E-125	246	289
63	26_C12	FM878159.1	Aar71	1	1	18	328152	1.30E-51	147	218
64	26_D02	FM878160.1	Aar72	1	1	3	6504564	7.00E-33	134	174
65	26_D03	FM878161.1	Aar73	1	1	1	86241856	7.10E-58	229	314
66	26_D05	FM878162.1	Aar74	1	1	Z	63109404	3.50E-59	133	192
67	26_D06	FM878163.1	Aar75	1	1	1	112205779	4.00E-63	146	245
68	26_D08	FM878164.1	Aar76	1	1	1A	21179116	3.00E-90	225	272
69	26_D10	FM878165.1	Aar77	1	1	3	81261081	3.20E-108	204	292
70	26_D12	FM878166.1	Aar78							
71	26_E01	FM878167.1	Aar79	1	1	1	70588689	1.60E-96	197	264
72	26_E02	FM878168.1	Aar80							
73	26_E03	FM878169.1	Aar81	2	1	6	26869936	3.00E-35	144	168
73	26_E03	FM878169.1	Aar81			6	26876850	2.40E-34	144	168
74	26_E04	FM878170.1	Aar82							
75	26_E06	FM878171.1	Aar83	1	1	2	126962817	1.50E-47	147	228
76	26_E11	FM878172.1	Aar84	2	2	10	16879330	2.30E-68	133	180
76	26_E11	FM878172.1	Aar84			Un	87827184	3.80E-62	131	180
77	26_F01	FM878173.1	Aar85	2	2	3	11316725	1.60E-67	237	247
77	26_F01	FM878173.1	Aar85			Un	31126367	6.40E-68	239	247
78	26_F02	FM878174.1	Aar86	2	2	1A	7762272	2.30E-62	188	289
78	26_F02	FM878174.1	Aar86			Un	8298542	1.40E-61	192	289
79	26_F05	FM878175.1	Aar87	2	2	4	63061310	7.40E-64	242	302
79	26_F05	FM878175.1	Aar87			Un	45830658	4.50E-63	240	302
80	26_F11	FM878176.1	Aar88	1	1	1	72887978	6.00E-71	276	377
81	26_G01	FM878177.1	Aar89	1	1	9	19809751	3.90E-82	257	344
82	26_G02	FM878178.1	Aar90	1	1	1	2907364	3.20E-103	280	416
83	26_G04	FM878179.1	Aar91	1	1	2	37026659	1.50E-101	376	427
84	26_G06	FM878180.1	Aar92							
85	26_G08	FM878181.1	Aar93	1	1	3	32921220	2.60E-21	97	145
86	26_G11	FM878182.1	Aar94	1	1	4	44229627	1.10E-96	231	295
87	26_H04	FM878183.1	Aar95	2	2	2	52306504	1.20E-75	296	377
87	26_H04	FM878183.1	Aar95			Un	133064525	5.20E-79	302	377
88	26_H06	FM878184.1	Aar96	1	1	3	87265586	1.20E-42	118	159
89	26_H08	FM878185.1	Aar97	1	1	1	23316476	2.30E-27	128	157
90	26_H09	FM878186.1	Aar98	1	1	3	91263342	3.40E-24	87	107
91	27_A05	FM878187.1	Aar99	1	1	2	45299624	7.60E-55	161	195
92	27_A07	FM878188.1	Aar100	1	1	5	32712638	1.40E-44	146	222
93	27_A10	FM878189.1	Aar101	1	1	Z	61000952	9.40E-18	90	103
94	27_B02	FM878190.1	Aar102							
95	27_B04	FM878191.1	Aar103	1	1	7	2735923	1.80E-72	228	232
96	27_B06	FM878192.1	Aar104	2	2	4	9675214	2.20E-33	134	158
96	27_B06	FM878192.1	Aar104			Un	9830441	7.40E-34	136	158
97	27_B07b	FM878193.1	Aar105	1	1	2	100440436	1.20E-98	333	509
98	27_B09	FM878194.1	Aar106	1	1	5	1054380	6.90E-78	177	232
99	27_B12	FM878195.1	Aar107	1	1	8	15112931	1.90E-50	161	218
100	27_C01	FM878196.1	Aar108	1	1	24	234487	3.90E-33	142	181
101	27_C02	FM878197.1	Aar109	1	1	1	72780799	8.30E-104	322	468
102	27_C05	FM878198.1	Aar110	1	1	2	38628781	7.10E-41	174	274
103	27_C07	FM878199.1	Aar111	1	1	17	3227358	2.40E-79	120	124
104	27_C09	FM878200.1	Aar112	1	1	5	42151037	2.60E-116	409	644
105	27_C10	FM878201.1	Aar113	2	2	1A	14268544	1.80E-109	273	352
105	27_C10	FM878201.1	Aar113			Un	24206086	9.30E-111	278	352
106	27_C11	FM878202.1	Aar114							
107	27_D01	FM878203.1	Aar115	1	1	13_random	1500058	5.40E-40	163	21
108	27_D03	FM878204.1	Aar116	1	1	2	153399249	7.00E-57	140	188
109	27_D10	FM878205.1	Aar117	1	1	4	39851603	1.60E-57	184	227
110	27_E01	FM878206.1	Aar118							
111	27_E02	FM878207.1	Aar119	1	1	3	82776170	2.90E-87	326	454
112	27_E06	FM878208.1	Aar120	1	1	Z	43145810	4.30E-24	111	182

113	27_E07	FM878209.1	Aar121	1	1	3	58597279	8.10E-68	159	316
114	27_E09	FM878210.1	Aar122	1	1	6	31599733	1.10E-12	68	85
115	27_F03	FM878211.1	Aar123	2	1	6	106327	1.70E-64	164	229
115	27_F03	FM878211.1	Aar123			6	114183	8.40E-58	138	234
116	27_F07	FM878212.1	Aar124	1	1	3	68194138	9.10E-82	187	216
117	27_F10	FM878213.1	Aar125	1	1	3	20725186	7.60E-88	336	436
118	27_G04	FM878214.1	Aar126	1	1	3	42629053	1.60E-53	213	250
119	27_G06	FM878215.1	Aar127	1	1	1	49913526	1.20E-99	211	276
120	27_G07	FM878216.1	Aar128	1	1	3	75947358	2.90E-103	203	287
121	27_G08b	FM878217.1	Aar129	2	2	1	38125037	1.10E-61	223	292
121	27_G08b	FM878217.1	Aar129			Un	99820199	1.80E-60	223	304
122	27_H08	FM878218.1	Aar130	1	1	2	49274114	4.20E-26	109	148
123	27_H11	FM878219.1	Aar131							
124	27_H12	FM878220.1	Aar132	1	1	3	85993527	2.80E-71	223	271
125	28_A06	FM878221.1	Aar133	1	1	Z	22817318	4.20E-73	238	370
126	28_A07	FM878222.1	Aar134							
127	28_B02	FM878223.1	Aar135	1	1	5	17449268	4.80E-79	305	499
128	28_B04	FM878224.1	Aar136							
129	28_B06	FM878225.1	Aar137							
130	28_B08	FM878226.1	Aar138	1	1	3	63078555	6.80E-74	240	360
131	28_B10	FM878227.1	Aar139	2	1	12	12355308	1.50E-47	114	123
131	28_B10	FM878227.1	Aar139			12	12352419	5.90E-47	112	123
132	28_B12	FM878228.1	Aar140	1	1	7	31491525	4.50E-44	116	150
133	28_C01	FM878229.1	Aar141	1	1	Z	13548109	8.00E-105	248	410
134	28_C03	FM878230.1	Aar142	1	1	4	38048181	6.40E-144	352	411
135	28_C05	FM878231.1	Aar143	1	1	8	1604904	3.00E-60	133	230
136	28_C08	FM878232.1	Aar144	1	1	2	147855609	1.20E-58	230	562
137	28_C09	FM878233.1	Aar145	1	1	26	2578572	5.30E-62	236	332
138	28_C10	FM878234.1	Aar146	2	2	7	28339875	3.00E-85	256	312
138	28_C10	FM878234.1	Aar146			Un	73081911	1.10E-83	256	312
139	28_C12	FM878235.1	Aar147							
140	28_E04	FM878236.1	Aar148	1	1	2	94298311	2.90E-82	230	343
141	28_E05	FM878237.1	Aar149							
142	28_E09	FM878238.1	Aar150	1	1	4	37235907	2.10E-129	471	664
143	28_E11	FM878239.1	Aar151	1	1	3	43753567	3.40E-58	142	172
144	28_F03	FM878240.1	Aar152	2	2	1A	38909898	2.10E-103	377	566
144	28_F03	FM878240.1	Aar152			Un	174970140	2.10E-103	377	566
145	28_G01	FM878241.1	Aar153							
146	28_G02	FM878242.1	Aar154							
147	28_G04	FM878243.1	Aar155	1	1	3	3250337	8.50E-116	340	395
148	28_G09	FM878244.1	Aar156	1	1	27	2040377	4.00E-93	342	456
149	28_G11	FM878245.1	Aar157	1	1	2	7034086	5.10E-126	355	485
150	28_G12	FM878246.1	Aar158	1	1	2	73327265	9.80E-105	287	351
151	28_H01	FM878247.1	Aar159	1	1	3	21425185	3.80E-96	361	474
152	28_H07	FM878248.1	Aar160	2	2	1	110144292	1.20E-59	209	307
152	28_H07	FM878248.1	Aar160			Un	160555905	6.80E-60	207	309
153	29_A02	FM878249.1	Aar161	2	1	19	4665090	1.10E-55	129	182
153	29_A02	FM878249.1	Aar161			19	4669315	1.10E-55	110	177
154	29_A03	FM878250.1	Aar162							
155	29_A06	FM878251.1	Aar163	1	1	11	7678737	4.70E-136	425	439
156	29_A11	FM878252.1	Aar164	1	1	2	65767247	4.20E-77	304	384
157	29_B01	FM878253.1	Aar165	2	2	24	3862869	2.70E-23	70	129
157	29_B01	FM878253.1	Aar165			Un	57151022	8.00E-19	61	130
158	29_B07	FM878254.1	Aar166	2	1	2	120639789	6.80E-113	419	583
158	29_B07	FM878254.1	Aar166			2	120536256	1.50E-110	411	586
159	29_B12	FM878255.1	Aar167	1	1	2	112183915	2.60E-20	101	208
160	29_C01	FM878256.1	Aar168	1	1	7	26103167	6.70E-120	300	422
161	29_C04	FM878257.1	Aar169							
162	29_C07	FM878258.1	Aar170	1	1	Z	41196678	2.80E-83	193	294
163	29_C10	FM878259.1	Aar171	2	2	2	53417994	1.30E-73	292	363
163	29_C10	FM878259.1	Aar171			Un	38072360	1.90E-78	292	363
164	29_D02	FM878260.1	Aar172	1	1	1	47599384	1.80E-35	111	199
165	29_D03	FM878261.1	Aar173	1	1	2	135836899	5.20E-126	312	376
166	29_D06	FM878262.1	Aar174	1	1	1A	35687229	1.90E-68	142	195
167	29_D07	FM878263.1	Aar175							
168	29_D09	FM878264.1	Aar176	1	1	2	40785262	7.70E-69	206	292
169	29_E03	FM878265.1	Aar177	2	1	2	45061284	2.10E-57	177	246

169	29_E03	FM878265.1	Aar177			2	45065312	3.00E-56	173	246
170	29_E08	FM878266.1	Aar178	1	1	3	76392648	5.00E-82	313	409
171	29_E09	FM878267.1	Aar179	1	1	7	30760030	4.90E-158	570	667
172	29_E12	FM878268.1	Aar180	1	1	18	9729266	9.10E-37	105	195
173	29_F01	FM878269.1	Aar181							
174	29_F03	FM878270.1	Aar182	1	1	1A	43479781	4.90E-70	246	343
175	29_F04	FM878271.1	Aar183	2	1	1A	54500979	3.70E-39	134	209
175	29_F04	FM878271.1	Aar183			1A	54497127	7.20E-39	133	210
176	29_G02	FM878272.1	Aar184	1	1	1	29117117	4.00E-31	96	118
177	29_G03	FM878273.1	Aar185							
178	29_G07	FM878274.1	Aar186	1	1	1A	43476341	4.90E-78	304	418
179	29_G09	FM878275.1	Aar187	1	1	11	15526307	6.70E-150	516	569
180	29_H09	FM878276.1	Aar188							
181	29_H11	FM878277.1	Aar189	1	1	1A	55540639	4.20E-44	168	216
		AJ276375.1	Ase18			3	13906080	5.90E-28	85	87
		AM072445.1	Calex-01			1A	45578197	1.30E-40	122	129
		AY769692.1	DkiD126_ZEST			1	4701224	5.10E-46	46	108
		DV955012.1	Tgu03			7	9322153	9.10E-141	355	355
