# Supplementary material for: Hybridization but No Evidence for Backcrossing and Introgression in a Sympatric Population of Great Reed Warblers and Clamorous Reed Warblers
Source: PLoS One. 2012 Feb 27;7(2):e31667. doi: 10.1371/journal.pone.0031667 (PMC3288047; doi:10.1371/journal.pone.0031667)
Supplement: Table S2 — Characteristics of 181 great reed warbler ( Acrocephalus arundinaceus ) microsatellite loci isolated and characterised in this study. Primer sequences, expected and observed product size, melting (T m) and annealing (T a) temperatures, 5′-fluoro-label, and amplification success in four test individuals are shown. (PDF) [file pone.0031667.s002.pdf]

Table S2 Characteristics of 181 great reed warbler (*Acrocephalus arundinaceus*) microsatellite loci isolated and characterised in this study. Primer sequences, expected and observed product size, melting (T_m) and annealing (T_a) temperatures, 5'-fluoro-label, and amplification success in four test individuals are shown.

Locus name	Accession number	Repeat motif	Motif length	Motif start	Motif end	Sequence length	Exp prod size	Tm	Label	Ta	Mono/ Poly	Observed fragment length
Aar9	FM878097.1	TTTC	21	405	488	570	374	65.2				
	Aar9F	ACCTTCATGCTGATGAAATAATTGTGG										
	Aar9R	GGGCCACTAACCTGGAAAGACAG										
Aar10	FM878098.1	TT	5	235	244	352	297	65.2	Hex	63	P	280-363
	Aar10F	TCTTCTCCATAAGCTCCATGTCTGG										
	Aar10R	CCGAGGAGCACCCACACTTC										
Aar11	FM878099.1	TTTT	5	465	484	632	287	63.9	Fam	62	M	292
	Aar11F	CATTAAAGCACAGGAGGTTTGTGG										
	Aar11R	AAGCCCTGGAGTACATTGGCTAAG										
Aar12	FM878100.1	TTTC	9	101	136	389	337	65.0				
	Aar12F	GCATTGGTTCTATGAAGGAGAATTAACG										
	Aar12R	AAGGGCAAAGCCTTCTGTATCTGAG										
Aar13	FM878101.1	AG	9	91	108	393	288	60.9				
	Aar13F	GCTTAGCTATCAAATGATTAAAGGAAAA										
	Aar13R	TCCACGTGAAGTAAGTGAACTGAA										
Aar14	FM878102.1	AAGAA	30	226	375	637	395	65.5				
	Aar14F	CCTCCAGACACTGCGCTTGATAC										
	Aar14R	TCCTGAGTTTCCCAACTATGCTTACG										
Aar15	FM878103.1	GA	8	155	170	391	263	60.8				
	Aar15F	TGTAGGAATATTTGTTTCCCAGAATC										
	Aar15R	ACATTAACTCCATCAAGCTCACATC										
Aar16	FM878104.1	TGGGA	5	31	55	409	NA	NA				
	Aar16F	NA										
	Aar16R	NA										
Aar17	FM878105.1	AA	5	161	170	556	400	63.2				
	Aar17F	GCACTGGGTGAGGGAATATGAG										
	Aar17R	CTTGGAGGTTTCACACTCTGCAC										
Aar18	FM878106.1	AA	5	165	174	726	288	64.8	Fam	63	P	259-288
	Aar18F	GCCACCACACAAGAAGTACATTGG										
	Aar18R	CTCCTCGCCACAGCTGACTG										
Aar19	FM878107.1	TGTG	7	253	280	416	218	63.3	Fam	61	P	218-223
	Aar19F	TGAGCTGATATCCAGATGATGTGTG										
	Aar19R	TTCCTAGGCAATTTATGCCCATAGT										
Aar20	FM878108.1	TCTA	11	1	44	432	NA	NA				
	Aar20F	NA										
	Aar20R	NA										
Aar21	FM878109.1	TTCTT	16	106	185	482	271	63.2				
	Aar21F	TCCTGGAAAGCTGAGTTTATACTGTTG										
	Aar21R	TGGTAAAGTGCCATTGCTACCAG										
Aar22	FM878110.1	GAAA	35	405	544	628	350	64.7				
	Aar22F	TTCAGCCTTGGTCTGCATTACG										

	Aar22R	AGCAACTGCATGGAACTTGTGC										
Aar23	FM878111.1	GAAA	30	109	228	554	500	60.7				
	Aar23F	GATGTTCTGGTGAATCAATAAAGTAGAGT										
	Aar23R	AAGAAGGGAAATACACCAATTATTTTCT										
Aar24	FM878112.1	TCTT	13	317	368	563	380	63.9				
	Aar24F	AATGTCTGTGTTACCTGGAGCACTTC										
	Aar24R	TTGAACAGAGTAAATGAGAAGGCATTG										
Aar25	FM878113.1	GGAA	25	161	260	638	559	64.6				
	Aar25F	TGAGGGTCATGATGGCTGATG										
	Aar25R	TGAAGAGTTGGTATTAAGGAGAATTCAAGC										
Aar26	FM878114.1	TATT	6	661	684	805	233	64.3				
	Aar26F	GCATTGAGTCGAAATACTGCATGTAAG										
	Aar26R	TTCAGGGTCCAAAGCCAACC										
Aar27	FM878115.1	GT	6	121	132	420	220	64.4	Hex	62	P	220-226
	Aar27F	CAGAAAGGAGAATTGGGATGCAC										
	Aar27R	CATTCATAGGAGTGCAACTCTCAAGC										
Aar28	FM878116.1	AAGG	20	149	228	536	519	60.3				
	Aar28F	CCCCTTTTGTCTGTAACTAACTATTGA										
	Aar28R	TCTCCTCCTCTATTCAATAAGCAATC										
Aar29	FM878117.1	TAGA	11	449	492	642	342	64.9				
	Aar29F	TTTGGAAGGACCACATACAGAGAAGTC										
	Aar29R	TGTGCATGGGACATGGTGTG										
Aar30	FM878118.1	TTCT	13	277	328	522	297	65.0				
	Aar30F	GCACTTGATGTAAACGCCTTGTCTT										
	Aar30R	TGGTCTCCAGGCAGAACCAAA										
Aar31	FM878119.1	TGTG	10	269	308	610	262	65.7				
	Aar31F	GGCTGGGAAGAATCCCATGATAAG										
	Aar31R	GGGCTAATGTTTAGGCCATTTGTTTC										
Aar32	FM878120.1	AAAG	16	389	452	746	487	60.8				
	Aar32F	AGGTCCTTATAAACACAAGAGAGCTG										
	Aar32R	AAACATTTAGAAAGCATCCAGCAG										
Aar33	FM878121.1	GATA	14	69	124	252	230	60.3	Hex	58	P	223-243
	Aar33F	TTTCAGTTGGAAAGATTTCAGAGAG										
	Aar33R	ACATTGGCAGTATGAAGCATACAC										
Aar34	FM878122.1	TCTA	11	21	64	485	NA	NA				
	Aar34F	NA										
	Aar34R	NA										
Aar35	FM878123.1	TT	6	91	102	377	186	64.4				
	Aar35F	TCGACCAGACCATCTCATTTGG										
	Aar35R	ACCAGTGAAAGACTCCAAGTCAGAAAC										
Aar36	FM878124.1	TTTC	9	329	364	599	385	63.4	Fam	61	P	323-465
	Aar36F	TTTGCCATTTGGAAAGCACAG										
	Aar36R	ATGGGCAAGGCAAAGAAAGG										
Aar37	FM878125.1	AA	6	57	68	525	181	64.9				
	Aar37F	AAAACCAAAATGCTCTCCGAAGG										
	Aar37R	CAGAGCACCTTTCTTTGGGCTTT										
Aar38	FM878126.1	AA	5	67	76	337	250	64.3	Hex	62	M	250
	Aar38F	TCAAGTCACTCCTTTACATCTCAAAAGC										
	Aar38R	GTGCCCACACAAGCAGCATT										
Aar39	FM878127.1	AGAG	9	105	140	533	237	63.9	Fam	62	P	216-237

	Aar39F	AAGCGGATAATAAACAGAGAGCATTTG										
	Aar39R	GCCAATCCCGAACAGGAAAC										
Aar40	FM878128.1	AAGG	19	21	96	451	NA	NA				
	Aar40F	NA										
	Aar40R	NA										
Aar41	FM878129.1	GATG	20	277	356	360	NA	NA				
	Aar41F	NA										
	Aar41R	NA										
Aar42	FM878130.1	AAAG	12	497	544	823	388	63.8				
	Aar42F	GAAACCAGATTGTTTGGGTTTATGC										
	Aar42R	AACAACCCAGGAGCAGACACTG										
Aar43	FM878131.1	GG	5	383	392	496	235	63.8				
	Aar43F	TGAACAGCAACTATGCTTTATATTCCTCTC										
	Aar43R	TGGTGATATAATTAATGTCTCTCCATCACTC										
Aar44	FM878132.1	ATGA	6	113	136	474	190	63.5				
	Aar44F	CAGTCCAGCTTTGAGCTCCTTG										
	Aar44R	TTTCCCACCCTGTGGAGTTG										
Aar45	FM878133.1	AC	7	149	162	666	239	65.2	Fam	63	M	239
	Aar45F	GGCATTTACTGTGCTCCATCCAC										
	Aar45R	GGGCAATTCATTTCCATTGGTG										
Aar46	FM878134.1	AC	5	95	104	425	192	63.4				
	Aar46F	GGGCCTCAGGAGAACTCCAC										
	Aar46R	AATGCTGTTTAGCATTAACATTGTTGG										
Aar47	FM878135.1	TCTC	6	173	196	621	292	63.5	Hex	62	P	290-304
	Aar47F	CTGTGGAGGCAGAGGGAAGAG										
	Aar47R	GGAAATGAACTGAGGATTTACACACAG										
Aar48	FM878136.1	ATCA	5	177	196	388	229	65.0				
	Aar48F	TGTCATTATGAGCTTGTTTATAGAAGGATATGG										
	Aar48R	TGTGAGCAATTCTTCTCTTGAATTGG										
Aar49	FM878137.1	TCTTT	29	61	205	513	NA	NA				
	Aar49F	NA										
	Aar49R	NA										
Aar50	FM878138.1	GTGT	5	161	180	445	202	63.3				
	Aar50F	GTGTGTATTTGAGGAAGGGAGAAGG										
	Aar50R	AATTCCCACAGGTGATTGTTGG										
Aar51	FM878139.1	TCTT	29	97	212	453	394	64.4				
	Aar51F	GCAGTTCTCTCTCCATGGTATTTGC										
	Aar51R	AAATCACCAGTGTGGGCATCC										
Aar52	FM878140.1	TTCT	11	89	132	285	NA	NA				
	Aar52F	NA										
	Aar52R	NA										
Aar53	FM878141.1	AA	5	353	362	538	457	64.9				
	Aar53F	AGGTTGGACCTTCCCAGTTTCAG										
	Aar53R	GATCCACAGGATTTGGGAAAGATG										
Aar54	FM878142.1	AAGAA	8	251	290	440	290	62.0				
	Aar54F	TGCTGAGCAAAATTAAAGCCTTC										
	Aar54R	TCGTGTTCAGTTACTTTTGATATATTCTCC										
Aar55	FM878143.1	TTTC	7	41	68	237	NA	NA				
	Aar55F	NA										
	Aar55R	NA										

Aar56	FM878144.1	ATTT	6	177	200	392	235	63.2				
	Aar56F	GGGAACTTGGTGGTACTTTGAGG										
	Aar56R	ACCAATGAGTGTTAACAGGTAAGAGAGAAG										
Aar57	FM878145.1	TATC	16	77	140	575	292	60.5	Fam	58	P	250-297
	Aar57F	TCTGGTGTAAACGTCTCTTATTTGATT										
	Aar57R	CCATCTCATCACATCTGTCACAC										
Aar58	FM878146.1	CAA	6	46	63	429	165	60.4	Fam	58	P	150-165
	Aar58F	TCGGTAACCAAAACCCAAAA										
	Aar58R	TTTAGAAAGGGCATCCCTGAG										
Aar59	FM878147.1	TATC	13	477	528	714	283	65.4	Fam	63	P	274-283
	Aar59F	TCCCTAGATGCTTCTGCTTTCTTCTG										
	Aar59R	TGAAGGTTGCACATTCCACAGG										
Aar60	FM878148.1	TCTC	7	341	368	381	NA	NA				
	Aar60F	NA										
	Aar60R	NA										
Aar61	FM878149.1	TTTC	21	129	212	365	331	63.8				
	Aar61F	CCTCACCATAAATGTATCCAGTCACC										
	Aar61R	TTTGGAGGACATAGAAATGTCTTAAAGATG										
Aar62	FM878150.1	TT	7	69	82	254	159	66.7	Fam	65	P	158-163
	Aar62F	CATGGTCCCTTCCAACCCAAAC										
	Aar62R	AAGGCAGCCCAGGAATATAAATATGAAATC										
Aar63	FM878151.1	GA	8	381	396	620	372	63.9				
	Aar63F	AAGTGAGCACTCAGTGGATTGTGAC										
	Aar63R	CTGTTCCTGACACTCTTCCTGTCC										
Aar64	FM878152.1	TAGGA	34	141	310	396	341	61.2				
	Aar64F	TTGGTTGCAATTTCCAAAGAAC										
	Aar64R	GATCAGTCCTGAAGTAGTCAGGTTGT										
Aar65	FM878153.1	TA	5	93	102	635	216	62.2	Fam	60	P	216-224
	Aar65F	TCAGTAGATGAAATAATTTACCTTTTGTAGAATTT										
	Aar65R	CTTGGCGAATCCAGTTCACA										
Aar66	FM878154.1	AATAG	28	41	180	399	NA	NA				
	Aar66F	NA										
	Aar66R	NA										
Aar67	FM878155.1	TAGAA	39	186	380	526	NA	NA				
	Aar67F	NA										
	Aar67R	NA										
Aar68	FM878156.1	CTAT	11	285	328	387	249	61.1				
	Aar68F	CGGGCATGAGGGAAAGTAAT										
	Aar68R	ACCCAAATTATTCTATGAACCCAAA										
Aar69	FM878157.1	ATCA	8	121	152	449	219	64.5				
	Aar69F	TTACTGTGCTGTTAAGAGAATAAACAATAAATGG										
	Aar69R	GGAAGCACCCAAAGAGCTCAAG										
Aar70	FM878158.1	CA	6	299	310	663	268	63.4	Hex	61	M	268
	Aar70F	GGTTGGTGAATAATGGCTGTGG										
	Aar70R	CAGTCACAATGCAACTGATAACAACTC										
Aar71	FM878159.1	AA	5	35	44	749	NA	NA				
	Aar71F	NA										
	Aar71R	NA										
Aar72	FM878160.1	AAAGA	27	66	200	584	495	64.8				
	Aar72F	CACACCACAGCAAAATCAAGTGC										
	Aar72R	CAGACCGGGTACTTATTCTGTCCCTA										
Aar73	FM878161.1	AGAA	39	37	192	751	NA	NA				

	Aar73F	NA										
	Aar73R	NA										
Aar74	FM878162.1	AGAA	5	221	240	438	284	62.9				
	Aar74F	AGATTTGTAGCTATTGTATCACAGGCATC										
	Aar74R	CCCTCCTCTTCTCCTCGTAAGC										
Aar75	FM878163.1	TGTG	7	257	284	450	270	65.8				
	Aar75F	AGGCTCTCCTTTCACGTCCATATTTC										
	Aar75R	GGGTGGTAAATGGGTAAATGGGTATG										
Aar76	FM878164.1	TGAT	6	449	472	529	245	64.7				
	Aar76F	TGAAACCCTTGCTGACCTTGG										
	Aar76R	TTCCAGCCTGCACAACTTTCC										
Aar77	FM878165.1	TT	7	231	244	715	282	63.9				
	Aar77F	ACCAGAAACTTTCCGTCATTGAATC										
	Aar77R	GCCAGCCTTTAACCCATTTGAG										
Aar78	FM878166.1	AAAG	12	181	228	472	376	63.6	Hex	62	P	280-376
	Aar78F	TTGCTTTCTGCTTCCTTAGATGGAT										
	Aar78R	CCAAATTTGACATTCTTGCAATTTCT										
Aar79	FM878167.1	AC	9	265	282	573	246	63.5				
	Aar79F	ACCTTCAGAAACCCATCATGTCC										
	Aar79R	GCCACTCTAGCAAATTCTGTTACCAC										
Aar80	FM878168.1	AAAG	13	369	420	689	580	64.9				
	Aar80F	TGCAGGGAACAACAATAACAACAAAC										
	Aar80R	AAATCAGCCCTGCTGTTGTGG										
Aar81	FM878169.1	AGAG	5	289	308	520	386	64.5	Hex	62	P	348-386
	Aar81F	TCCATGGGAGGCAGTGTCTG										
	Aar81R	AACTGAGCACACATTCAGAACAGAGAG										
Aar82	FM878170.1	GAAAG	36	96	275	510	500	63.3				
	Aar82F	CCAGGGCTGGAACTCTTGG										
	Aar82R	TTGCCCTGAAAGGAAGGATTG										
Aar83	FM878171.1	CTAT	5	117	136	390	288	64.4				
	Aar83F	GGTATCAGATTGTGGAAGGTGAAGC										
	Aar83R	ATGGCAACCTGTGCAGCATC										
Aar84	FM878172.1	AA	5	287	296	628	375	66.2	Fam	64	P	356-456
	Aar84F	TGGCACTCTCAGTGTGGCTTTG										
	Aar84R	GCTAGCAGGGCTGCACATGG										
Aar85	FM878173.1	TCTT	24	249	344	435	288	60.5				
	Aar85F	CAAGAGCATGAAGCTGGAGAA										
	Aar85R	TCAGCAATTTCTTATTCTAAAATCCTCT										
Aar86	FM878174.1	TCTA	6	113	136	582	278	64.9	Hex	63	P	278-302
	Aar86F	GTGAGCTCCAGGAAGGCACTG										
	Aar86R	TCTAGTAGGAAGCCTGAATGACCACTG										
Aar87	FM878175.1	TT	5	51	60	387	233	63.8				
	Aar87F	TTCTGTAGAGTCATCTCTCTGTCACCTG										
	Aar87R	GCAGATTTAATTGCAGATAATGAAGTATGG										
Aar88	FM878176.1	AGCT	9	241	276	711	395	64.2				
	Aar88F	GCTTAGCCAATCAAGGGACACC										
	Aar88R	GCAGGGTCTAGGAAAGCCAATG										
Aar89	FM878177.1	AC	6	89	100	565	237	63.7				
	Aar89F	GGAAATATTCCGCTGACACATCC										
	Aar89R	TGGCTCTAAATCAATCAATCAATCATC										
Aar90	FM878178.1	AA	5	555	564	604	182	63.9	Fam	62	NA	
	Aar90F	GGTGGCTTCACAACTTTGTTGC										
	Aar90R	CGCTCTGGAGCTGTGCTTTC										
Aar91	FM878179.1	TTTC	36	429	572	640	NA	NA				
	Aar91F	NA										
	Aar91R	NA										
Aar92	FM878180.1	AAGG	22	141	228	359	278	64.3				
	Aar92F	CAGCATGATACATGGCTCAGAGG										
	Aar92R	TGCCTCACCACTTCATGGTTG										

Aar93	FM878181.1	CTTT	9	197	232	521	417	63.6				
	Aar93F	TCATCAACAAACTTTGGCTCTGG										
	Aar93R	GGCAAGAAAGAGCGCAAGAAG										
Aar94	FM878182.1	TG	8	343	358	584	344	63.9				
	Aar94F	AGCTGAATTAGTTCCCACACATCATC										
	Aar94R	CCAAGCAGAATGTGTGAAACAGG										
Aar95	FM878183.1	AGAG	7	401	428	443	NA	NA				
	Aar95F	NA										
	Aar95R	NA										
Aar96	FM878184.1	AACA	6	181	204	387	225	63.4				
	Aar96F	AGTGAGATTGAAGAAATGTCCACAGAC										
	Aar96R	TGACACGTGAAGAGGTTCATTCC										
Aar97	FM878185.1	GA	5	703	712	720	NA	NA				
	Aar97F	NA										
	Aar97R	NA										
Aar98	FM878186.1	GGAA	30	113	232	395	328	64.4				
	Aar98F	CTTTCAAAACTATGGTATATGATTCCAAAGAAA										
	Aar98R	TGCCTTTCATTCCTTCCATTCC										
Aar99	FM878187.1	TATC	14	105	160	371	295	64.7				
	Aar99F	GGGCTTTCAGAGGTGGAGACC										
	Aar99R	TGTCCTGAGGAAATGCTTCCACTAC										
Aar100	FM878188.1	GA	5	193	202	437	329	63.7				
	Aar100F	CGGAAGTGCTTTGAGGAACTAAGTC										
	Aar100R	TCACTTCCAAAGCCTAAATCAAAGG										
Aar101	FM878189.1	GAAA	24	161	256	451	384	64.4				
	Aar101F	CTCACCAGGTTAGAGAAACGGAATG										
	Aar101R	CCTTTCACTGCTGTTTACCAGCTTC										
Aar102	FM878190.1	TTCTC	25	26	150	304	NA	NA				
	Aar102F	NA										
	Aar102R	NA										
Aar103	FM878191.1	ATAG	9	421	456	529	196	63.7	Hex	62	P	190-196
	Aar103F	CGAGCCTTTACTCAGAACATCTGC										
	Aar103R	CATCATCATCATCCCTCCAAGTG										
Aar104	FM878192.1	TCT	5	151	165	574	563	63.1				
	Aar104F	GGCAGAGCCAAACACTTTGC										
	Aar104R	AGGCATCAGACATTGATTAAATAATAATAAGAAG										
Aar105	FM878193.1	GAAA	42	505	672	742	398	61.0				
	Aar105F	CCCAGCTACTTAATTGGGTTCC										
	Aar105R	GAACCATTGTCACACTCTTCTGG										
Aar106	FM878194.1	TCTA	7	269	296	533	495	65.2				
	Aar106F	GCTATTTCTTCTGTTCTAGCAATTGATACCC										
	Aar106R	AACAATTCTAACAAGGAACTGCCATAACC										
Aar107	FM878195.1	ACAC	5	221	240	348	198	62.3				
	Aar107F	TCAATCTCCCAGGCTGCAC										
	Aar107R	TTTACCTTAAGTTTCTATTTAACTCTACTGACAGG										
Aar108	FM878196.1	CTTT	16	57	120	332	317	61.6				
	Aar108F	TCGTGGGCCTTAACAACTTTAAC										
	Aar108R	AGCTGCCACACCTACAATGTATTC										
Aar109	FM878197.1	TAGA	13	521	572	714	294	63.5				
	Aar109F	TTGCTGCTGTGGTTACTGTCAAATAC										
	Aar109R	TCCTGGGACAAGAATCCCAAC										
Aar110	FM878198.1	TAGA	13	281	332	342	NA	NA				
	Aar110F	NA										
	Aar110R	NA										
Aar111	FM878199.1	TG	5	285	294	638	360	64.7	Hex	63	P	360-373
	Aar111F	TCAATTGGGAAGGTGGAGCAC										
	Aar111R	CCACAGCTGGAATTCCTCTCTCTC										
Aar112	FM878200.1	TG	8	79	94	740	237	64.3				
	Aar112F	TCTGGACTGTAGACAACAAATCTCTGAAG										

	Aar112R	AATCCGAGTAACCCAGAAATATGCAC										
Aar113	FM878201.1	TCTA	10	565	604	657	232	60.6				
	Aar113F	TGCTTGTGTTTGCTTCCGTA										
	Aar113R	GATCAGGGAGTTTCATGGATAATAAA										
Aar114	FM878202.1	TGAT	6	401	424	534	191	63.1				
	Aar114F	GACAGGTGTCATGAACAAATTCCTC										
	Aar114R	AATAACTGCCTAATTGAACTAGCACTGG										
Aar115	FM878203.1	GATA	13	45	96	310	262	63.4				
	Aar115F	ACAGAAGCCTTCAGCAGTGGAG										
	Aar115R	TTTAATATCCAAATAGTTACCTGGAGAATGATAAC										
Aar116	FM878204.1	TTCT	24	241	336	649	473	65.0				
	Aar116F	ACGTTGGATGAGTTTGAAGAGATGC										
	Aar116R	TCTGCAGTCAGGGAACAAGGAAG										
Aar117	FM878205.1	TCTT	6	281	304	561	395	64.0				
	Aar117F	TGGGAATTTCTTTAAGCCAGAAGC										
	Aar117R	CCAGACTGAAATTAATCAGTGGTTCTTATG										
Aar118	FM878206.1	AGGA	27	85	192	500	483	64.3				
	Aar118F	AGCACATGGCAGTGGGATTG										
	Aar118R	CATGTGAGCTGGGGCTGAAG										
Aar119	FM878207.1	CT	6	39	50	511	150	64.8	Fam	63	P	150-155
	Aar119F	TTCCATGTGCTGGTGCTGAAG										
	Aar119R	AATCAGGGCCTGCCAGAGG										
Aar120	FM878208.1	CTTT	24	193	288	473	NA	NA				
	Aar120F	NA										
	Aar120R	NA										
Aar121	FM878209.1	GAGA	6	149	172	528	224	64.7				
	Aar121F	GAGGCCTAGCTGAAGGGAAAGC										
	Aar121R	TGTTTGCTTGCTCTGGAATGATG										
Aar122	FM878210.1	GAAGA	6	86	115	354	NA	NA				
	Aar122F	NA										
	Aar122R	NA										
Aar123	FM878211.1	TTTCT	40	171	370	601	493	65.4				
	Aar123F	GCCTTTGTAGCCTCAACTAAGGAAGC										
	Aar123R	TTGGCAATAAGCAGCCAGTAACATTAG										
Aar124	FM878212.1	AA	5	317	326	643	273	63.3	Fam	61	P	160-273
	Aar124F	GTGTATCAGTGGTCGTCTGTGTCC										
	Aar124R	GCAATGGAGCTGCCTCTCTG										
Aar125	FM878213.1	TCTA	7	437	464	498	NA	NA				
	Aar125F	NA										
	Aar125R	NA										
Aar126	FM878214.1	AAGA	7	253	280	376	154	60.7				
	Aar126F	GTCTCCTCTCCTTGGGTTCAA										
	Aar126R	CTGAATACTCATGTGAAAGTCATGAAA										
Aar127	FM878215.1	TATT	6	285	308	540	361	65.0				
	Aar127F	TGCCTTTGCCTCTTAATCACTGAAG										
	Aar127R	GCCATATGAGACTGCTGCATGG										
Aar128	FM878216.1	TA	7	317	330	646	247	65.5				
	Aar128F	TGCCAACCCACACCATTCTG										
	Aar128R	CCACAACTGAATTTCATAGGCAACG										
Aar129	FM878217.1	TCTT	36	389	532	618	305	60.3				
	Aar129F	GGAGTTCTGGGATGCTATGGT										
	Aar129R	GATCCAAGATAAATCGTCTTAGAATAGACT										
Aar130	FM878218.1	GAAG	18	49	120	533	NA	NA				
	Aar130F	NA										
	Aar130R	NA										
Aar131	FM878219.1	AGAAA	21	76	180	544	437	61.2				
	Aar131F	TTGTGTACAATTAATGTAGTCAGACAATCTC										
	Aar131R	TGCTCTGCCATGTTTGATTTC										
Aar132	FM878220.1	ACAC	8	389	420	472	227	64.6	Fam	63	P	220-227

	Aar132F	GGAGAAGCTTGTTATCCCAAACCA										
	Aar132R	TGAGGATAGACTTAATTAAAAGTTGGAGTATTGG										
Aar133	FM878221.1	AC	5	361	370	562	279	63.5				
	Aar133F	AGAATGCCATTTGGTAGAAATCAATG										
	Aar133R	CTCCACTACAACAGAGAGGCGTATTC										
Aar134	FM878222.1	TC	5	689	698	739	195	64.4				
	Aar134F	TTCTCGGAACTTCTGCACCTCTC										
	Aar134R	TTTATGCTGCCAGCCACAGG										
Aar135	FM878223.1	CA	7	195	208	488	162	62.2				
	Aar135F	GGGACTATGTGGGCTGGGTA										
	Aar135R	TTTGTTTCTTCCTCTTTCAAATGAGAC										
Aar136	FM878224.1	TG	7	693	706	764	180	63.6				
	Aar136F	TCCACTGGCAACATTCACACTATTAC										
	Aar136R	TCATCCTTTCGGTCCCTTTCTC										
Aar137	FM878225.1	AG	7	193	206	390	243	65.6				
	Aar137F	TGGCACAAGATGAAGCTTTCAATG										
	Aar137R	TTTGCTTAAATCCACAAATAAACAATAATCTCC										
Aar138	FM878226.1	AT	5	405	414	546	223	63.2				
	Aar138F	CATGTTGAAGAAACTTGGTTGCAG										
	Aar138R	GATGACTAACTCGCCACAAGCAC										
Aar139	FM878227.1	ATCT	14	141	196	340	238	63.3				
	Aar139F	TGACTTCATTGCATGTTCAGCAC										
	Aar139R	TTGGGAGCACAGACATGTGG										
Aar140	FM878228.1	TAGA	17	21	88	346	NA	NA				
	Aar140F	NA										
	Aar140R	NA										
Aar141	FM878229.1	AAA	5	343	357	801	177	64.1	Hex	62	M	171
	Aar141F	ACCACAAGAGGGAGTTCCTTGC										
	Aar141R	TGTGTGTGTGTTTGCCTTTGTTG										
Aar142	FM878230.1	AAA	5	412	426	669	264	65.0	Hex	63	P	260-277
	Aar142F	GAAAGAGAACTCCAAATCACTCTGCTG										
	Aar142R	CAACAAGAATCCTCTGTTCCCTAATCC										
Aar143	FM878231.1	ACAA	7	337	364	605	258	65.0				
	Aar143F	TGAGGTGTTCTGGCACTGCAC										
	Aar143R	CCATTGTTCCAGGGAGTTCAGC										
Aar144	FM878232.1	AA	5	257	266	543	224	63.3	Hex	61	P	220-224
	Aar144F	ACACAGGCACACACACATACCC										
	Aar144R	GCACCTGCACTGAGTAACTTTGC										
Aar145	FM878233.1	TT	6	225	236	332	236	64.3	Hex	62	P	236-238
	Aar145F	AGAGAGCAGCAATGGCTCTGG										
	Aar145R	GGAGCGCCTGCAGTGAATATC										
Aar146	FM878234.1	GT	5	361	370	521	187	65.0				
	Aar146F	AGACACTCTGCCCACACACCTG										
	Aar146R	TGCCACTTCCAGAGCAGTTCC										
Aar147	FM878235.1	AAGAG	45	291	515	601	376	60.9				
	Aar147F	AGCAAGGCCTGTAGCAGTAGG										
	Aar147R	AAATAAATGTCCATCTACTCAGGTATTATGA										
Aar148	FM878236.1	GT	5	147	156	592	232	65.0	Fam	63	M	237
	Aar148F	TCCTGAGGCTGAGAGCAATGTG										
	Aar148R	GTCCTGTGGTTGATAATGGCAGTG										
Aar149	FM878237.1	AG	6	261	272	442	236	63.6				
	Aar149F	CAAAGGAGGCCAAGAGAATGC										
	Aar149R	TGGATTGCTGCGAGGACTTC										
Aar150	FM878238.1	TG	5	443	452	652	236	63.7				
	Aar150F	TGCTGGCCAGTGCTATCTAAATG										
	Aar150R	TCACTGCAGAGTTCCAATGGTTC										
Aar151	FM878239.1	AAAA	5	173	192	325	215	65.3				
	Aar151F	GGGCTGTCACGGACCTGAAC										
	Aar151R	TTGCCAACCTCCCACATTCC										

Aar152	FM878240.1	AC	9	517	534	563	NA	NA				
	Aar152F	NA										
	Aar152R	NA										
Aar153	FM878241.1	AAAA	5	73	92	310	212	63.5				
	Aar153F	TCATCTCCATTTTCTGTATTTTGATGTTC										
	Aar153R	TGGTGTTGGTGTGGGGTTTT										
Aar154	FM878242.1	CACA	5	337	356	363	NA	NA				
	Aar154F	NA										
	Aar154R	NA										
Aar155	FM878243.1	AC	6	173	184	582	190	64.7	Hex	63	M	189
	Aar155F	CCATTTATGCCTGATTATTTGGTCAAG										
	Aar155R	AACTGAGAACAGGCTTGGCAATG										
Aar156	FM878244.1	TT	5	165	174	513	218	64.5				
	Aar156F	TTACACACAGCCCTCCGTATGC										
	Aar156R	CATGTGCCCAAACCTTGGTG										
Aar157	FM878245.1	GA	5	49	58	656	151	65.5	Fam	63	M	150
	Aar157F	TGAAAGAAATGTTTGTTTTCTTGGGAAG										
	Aar157R	CAAGGCCACAGAGGCTCAGG										
Aar158	FM878246.1	TG	6	337	348	700	249	63.9	Hex	62	P	249-250
	Aar158F	CATGTGTGCATGTAGGAGTGTGC										
	Aar158R	ACTGCCAACAGTGCAGAGTGC										
Aar159	FM878247.1	AC	5	309	318	470	400	63.4				
	Aar159F	TGGGAAGCAACAACCAGACAC										
	Aar159R	TCATGACTGCACGACCTATTTCC										
Aar160	FM878248.1	ATAG	9	445	480	532	217	63.9				
	Aar160F	GTGTCAATGAGGCGGGTTTG										
	Aar160R	TTACCCATGGTTGATGATACCTTCC										
Aar161	FM878249.1	TTA	5	274	288	456	366	64.8				
	Aar161F	CCCAAACACCTCAGGGATGC										
	Aar161R	TACCTGCAGGCCCAGGGTAG										
Aar162	FM878250.1	GAGA	6	97	120	200	176	65.1				
	Aar162F	AGGGTGCAAATGTGAGACCATGT										
	Aar162R	ATCCACCGAGTGGGGAGGTT										
Aar163	FM878251.1	AC	5	135	144	584	247	65.4	Fam	63	M	247
	Aar163F	TGGGTCTGAATTGGGAATCACTG										
	Aar163R	AGCAAAGCTCTGGGCCTCTTTC										
Aar164	FM878252.1	TTTT	5	385	404	421	NA	NA				
	Aar164F	NA										
	Aar164R	NA										
Aar165	FM878253.1	CA	7	131	144	302	231	64.4				
	Aar165F	CAGTTGCCCAAGTGTAGTCAGACC										
	Aar165R	GCAGCATAAGGCCAGAACAGC										
Aar166	FM878254.1	TT	5	265	274	582	335	64.1				
	Aar166F	AAATCTCTTTGCTTTATTCTACACTAACATTGG										
	Aar166R	TGCACACCCACTCATACACACC										
Aar167	FM878255.1	GT	6	11	22	218	NA	NA				
	Aar167F	NA										
	Aar167R	NA										
Aar168	FM878256.1	TTTT	6	585	608	747	236	65.5	Hex	63	P	230-237
	Aar168F	TGACTCTGCCAATGACTGCACA										
	Aar168R	CACATACATTCCCTGGGGAAAATAAAA										
Aar169	FM878257.1	TT	5	155	164	292	198	63.8				
	Aar169F	AACTGGGTATTGGAAGAAGATGCTTTAG										
	Aar169R	TTTCCTCTAAAGAACTAATCAGAAGGAAACTAAC										
Aar170	FM878258.1	TTGTT	5	291	315	647	208	64.8				
	Aar170F	TGAGGACCCTCAAACCGAGTG										
	Aar170R	CTGCAGAGCAGGAGAGGGATG										
Aar171	FM878259.1	AG	6	309	320	364	NA	NA				
	Aar171F	NA										

	Aar171R	NA										
Aar172	FM878260.1	AA	5	201	210	327	222	62.0				
	Aar172F	AATGCCTGCCTCGTAGTCTCA										
	Aar172R	GATCTTATTTAAAGGCTTACTTGAAACAGAA										
Aar173	FM878261.1	TGT	5	253	267	643	**551**	65.2	Hex	58	P	303-309
	Aar173F	GGTGGCCAAATGGCTCTAAGG										
	Aar173R	TGAAAGCATGGCCCTGAAATG										
Aar174	FM878262.1	TC	8	69	84	649	178	65.2				
	Aar174F	GCAAGGAGATATGGCAGAGTAATGAAAG										
	Aar174R	TCCGCTTCTTCCTCCTTGACTG										
Aar175	FM878263.1	AG	7	215	228	258	NA	NA				
	Aar175F	NA										
	Aar175R	NA										
Aar176	FM878264.1	AGAG	9	361	396	701	456	64.3				
	Aar176F	CCAAAGACTTGATACATTGCTTCAGTG										
	Aar176R	CAGGGTTCCAGGGAGTCTGC										
Aar177	FM878265.1	TT	5	455	464	553	239	64.5	Fam	62	P	239-243
	Aar177F	AATTGCACCAGTCAAGGAACACTG										
	Aar177R	AGCATGTTGGGTCCATGCTG										
Aar178	FM878266.1	AAGG	28	37	148	706	NA	NA				
	Aar178F	NA										
	Aar178R	NA										
Aar179	FM878267.1	AC	7	413	426	663	207	64.4				
	Aar179F	GGAATTTCAAATGGGATTGTTTATGC										
	Aar179R	TTGTGTGTGACGTGGCATCTG										
Aar180	FM878268.1	TG	5	201	210	363	235	63.3				
	Aar180F	GGATGGAGGAAGGAAGGAAGG										
	Aar180R	CATTCTAACACCCTCTCTGCAAATG										
Aar181	FM878269.1	CTCTT	37	61	245	382	329	64.9				
	Aar181F	GGCAGCCTTGCCCTGCTA										
	Aar181R	CTGCCATCACGTTGGCTAATTTC										
Aar182	FM878270.1	AG	5	371	380	454	245	63.8				
	Aar182F	ATAAACCACTCGCGGGAAGC										
	Aar182R	TTCCTTGTTGCTGCTCTTGTGAG										
Aar183	FM878271.1	TT	5	59	68	546	239	64.9				
	Aar183F	TCATAACTGTTTGGTTGGGTTTGAGTT										
	Aar183R	GCTCGAGAGCTTCCCAGCAG										
Aar184	FM878272.1	GA	5	67	76	215	182	65.1	Hex	63	M	180
	Aar184F	CAGCATGTGCATACAAAGGTCTCC										
	Aar184R	CGGCACATGCAGCTTGAATC										
Aar185	FM878273.1	AG	6	185	196	365	272	65.3				
	Aar185F	GGCTCTAGCTGGCTCAGTTCCTC										
	Aar185R	TGCCATGTAGTTGGACCCTTCC										
Aar186	FM878274.1	AC	6	241	252	416	224	63.7				
	Aar186F	TTTCTTCAACTTACCCTGGCTGAAC										
	Aar186R	ATTCTGGAGTTCTAATCTTGAGAATTAGTGG										
Aar187	FM878275.1	ACAC	9	581	616	685	247	63.6	Hex	62	P	239-253
	Aar187F	TGCTGAAATGAAGGTGTCAAACTG										
	Aar187R	CCATGCATTAGAATGCCCATAAAC										
Aar188	FM878276.1	GAGA	8	77	108	615	223	64.4				
	Aar188F	GAGCCTCGTGGCAGGATAGTG										
	Aar188R	TTCGTTGTCTGTGTGCCATCC										
Aar189	FM878277.1	GAGA	5	45	64	303	195	65.1				
	Aar189F	TCCAGCATTGCATAGGTGTGAATAAC										
	Aar189R	TCAGCAGCACACTTTGCTAGGG										
