# Supplementary material for: Hybridization but No Evidence for Backcrossing and Introgression in a Sympatric Population of Great Reed Warblers and Clamorous Reed Warblers
Source: PLoS One. 2012 Feb 27;7(2):e31667. doi: 10.1371/journal.pone.0031667 (PMC3288047; doi:10.1371/journal.pone.0031667)
Supplement: Table S3 — Number of alleles, range of fragment length (bp), expected and observed heterozygosity, Wright's FIS, and two-tailed P -value for deviations of Hardy-Weinberg equilibrium ( P HW; shown when P <0.1) in great and clamorous reed warblers. Also given are null allele frequencies in the great reed warbler. (PDF) [file pone.0031667.s003.pdf]

Table S3 Number of alleles (N_A), range of fragment length (bp), expected and observed heterozygosity, Wright's F_{IS}, and two-tailed *P*-value for deviations of Hardy-Weinberg equilibrium (P_{HW}; shown when $P < 0.1$) in great and clamorous reed warblers. Also given are null allele frequencies (Null freq.) in the great reed warbler.

		Great reed warbler							Clamorous reed warbler					
**Locus name**	**Accession number**	N_A	**Range**	H_{EXP}	H_{OBS}	F_{IS}	P_{HW}	**Null freq.**	N_A	**Range**	H_{EXP}	H_{OBS}	F_{IS}	P_{HW}
Aar18	FM878106.1	26	255-327	0.96	0.96	0.00		-0.01	4	252-256	0.70	0.57	0.19	
Aar19	FM878107.1	2	221-222	0.43	0.61	-0.42		-0.38	4	215-221	0.71	0.47	0.34	0.088
Aar27	FM878115.1	2	224-226	0.49	0.54	-0.09		-0.06	2	224-226	0.07	0.07	0.00	
Aar33	FM878121.1	8	211-239	0.77	0.85	-0.11		-0.07	5	211-227	0.83	0.45	0.45	0.017
Aar36	FM878124.1	25	283-425	0.97	0.89	0.08		0.03	12	283-434	0.97	0.75	0.23	0.068
Aar39	FM878127.1	9	211-233	0.74	0.56	0.25	0.010	0.12	5	205-235	0.27	0.21	0.21	
Aar47	FM878135.1	10	290-304	0.83	0.71	0.13		0.07	10	290-300	0.64	0.73	-0.15	
Aar57	FM878145.1	9	250-294	0.83	0.82	0.01		0.00	9	245-284	0.89	0.55	0.39	0.008
Aar62	FM878150.1	6	159-164	0.76	0.68	0.10		0.04	5	163-167	0.61	0.60	0.02	
Aar84	FM878172.1	14	355-449	0.88	0.89	-0.01		-0.02	9	365-395	0.80	0.87	-0.08	
Aar86	FM878174.1	6	274-294	0.56	0.44	0.20		0.10	6	270-294	0.76	0.80	-0.05	
Aar111	FM878199.1	4	369-373	0.30	0.30	0.01		0.08	2	371-373	0.20	0.21	-0.08	
Aar119	FM878207.1	4	153-156	0.26	0.18	0.32	0.068	0.14	5	151-157	0.72	0.73	-0.02	
Aar142	FM878230.1	17	260-296	0.85	0.78	0.09		0.03	11	257-282	0.88	0.87	0.02	
Aar144	FM878232.1	5	221-225	0.72	0.68	0.06		0.02	4	219-222	0.58	0.60	-0.03	
Aar145	FM878233.1	2	236-237	0.43	0.39	0.09		0.03	2	235-236	0.07	0.07	0.00	
Aar158	FM878246.1	3	251-253	0.14	0.14	-0.04		-0.07	1	251	0.00	0.00	NA	
Aar173	FM878261.1	6	297-313	0.64	0.59	0.07		0.02	11	297-318	0.93	0.91	0.02	
Aar187	FM878275.1	10	231-253	0.86	0.89	-0.04		-0.03	3	233-241	0.42	0.53	-0.28	
Ase18[#]	AJ276375.1	3	163-175	0.17	0.18	-0.06		-0.09	8	165-186	0.79	0.62	0.22	
Calex-01[¤]	AM072445.1	3	223-227	0.43	0.50	-0.16		-0.10	1	225	0.00	0.00	NA	
DkiD126 Tgu EST[£]	AY769692.1	2	243-244	0.25	0.14	0.43		0.15	1	243	0.00	0.00	NA	
Tgu03[$]	DV955012.1	7	260-276	0.74	0.85	-0.15		-0.09	8	266-280	0.88	1.00	-0.14	

[#] Ase18F: ATCCAGTCTTCGCAAAAGCC; Ase18R: TGCCCCAGAGGGAAGAAG; Ta: 58; Richardson *et al.* (2000) Mol Ecol 9: 2226-2231

[¤] Calex-01F: CTTCTCCATTGTTGTCACCTCCAGT; Ase18R: GTTTCTTCTTGACTTGGCCTGAGGTTTAGGTT; Ta: 58; Kupper *et al.* (2007) Mol Ecol Notes 7: 35-39

[£] DkiD126 Tgu EST-F: ACCTGGCTGCCCCAGT; DkiD126 Tgu EST-R: TCAAGGCCAGGCTGGAT; Ta: 58; DA Dawson, unpublished; King *et al.* (2005) 5: 569-571

[$] Tgu03F: TCTCAGCATCAGTGTGTTCTGTAC; Tgu03R: TTTGCTTGGGATTGAAGTGA; Ta: 58; Slate *et al.* (2007) BMC Genomics 8: 52
