# Supplementary material for: Hybridization but No Evidence for Backcrossing and Introgression in a Sympatric Population of Great Reed Warblers and Clamorous Reed Warblers
Source: PLoS One. 2012 Feb 27;7(2):e31667. doi: 10.1371/journal.pone.0031667 (PMC3288047; doi:10.1371/journal.pone.0031667)
Supplement: Figure S1 — Chromosomal locations of great reed warbler ( Acrocephalus arundinaceus ) microsatellite loci and four other avian microsatellite loci included in the present study (Table S1) on the zebra finch ( Taeniopygia guttata ) genome. (PDF) [file pone.0031667.s004.pdf]

Supplementary Figure S1 Chromosomal locations of great reed warbler (*Acrocephalus arundinaceus*) microsatellite loci and four other passerine microsatellite loci included in the present study (Table S1) on the zebra finch (*Taeniopygia guttata*) genome*

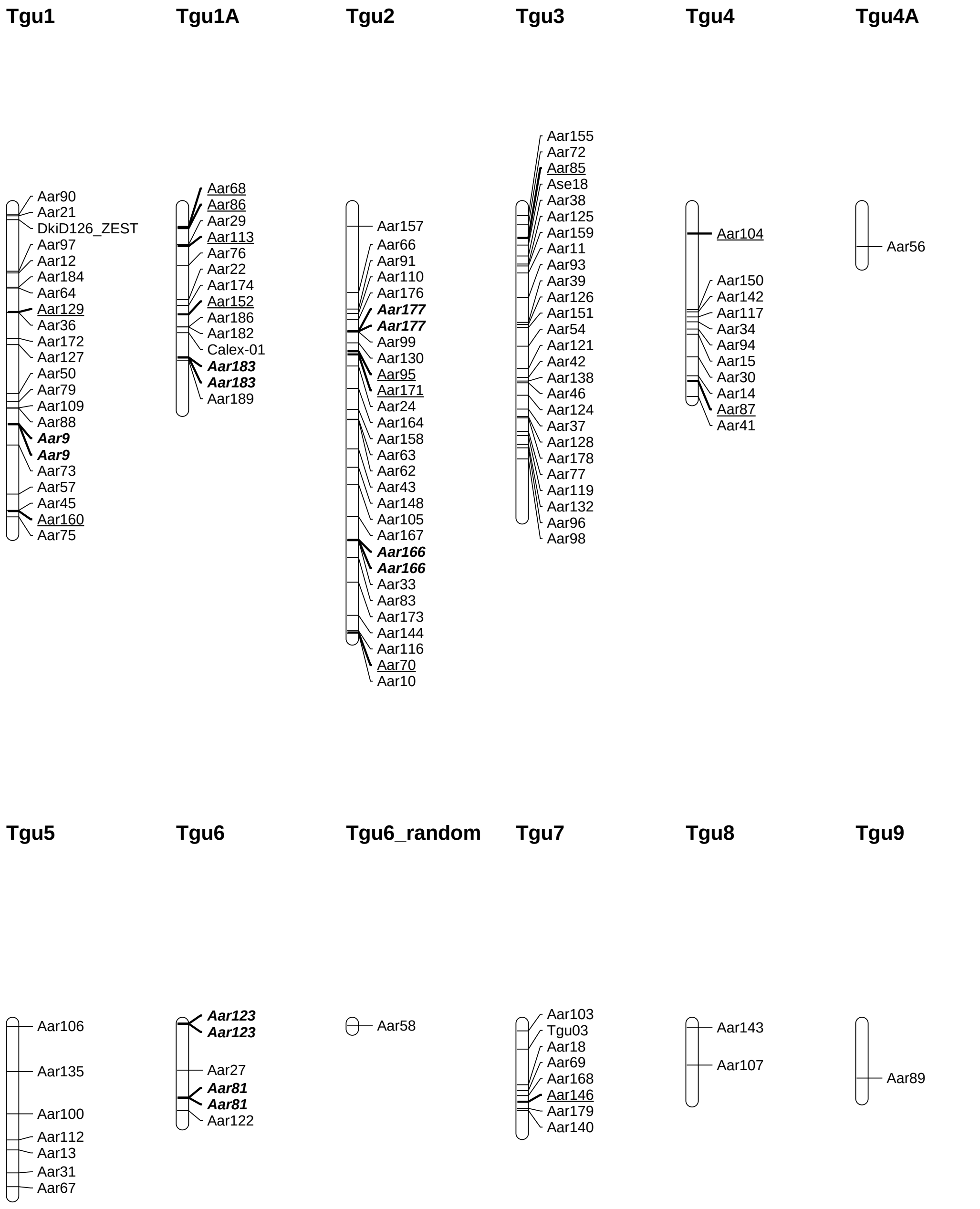

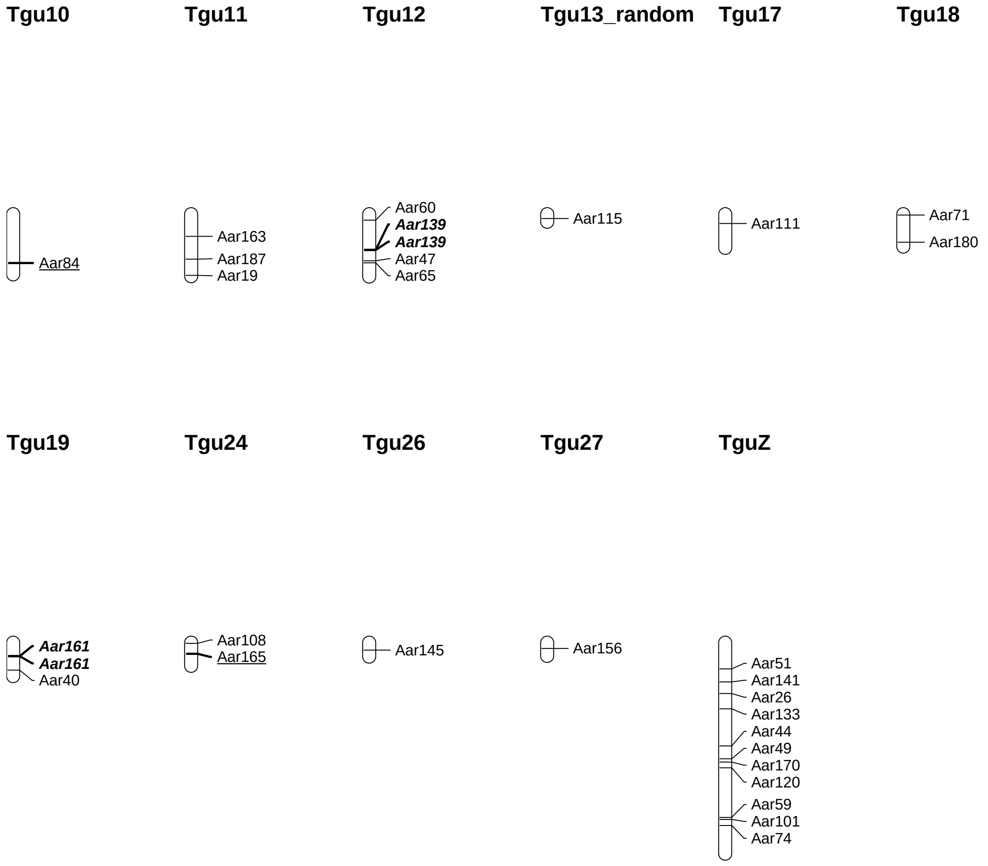

* Summary methods: Locations were assigned based on sequence homology the zebra finch genome assembly (tgu3.2.4, build 1.1) and identified using BLAST analyses (E-value < 1E-10) implemented on the ENSEMBL webpage http://www.ensembl.org/Multi/blastview; Search tool: BLASTN; Search sensitivity: Distant homologies; Single hit criteria: E-value <1E-10; a factor of 1E-10 lower, or a score 100 higher, than second hit; those loci displaying two hits (not matching the single hit criteria) are shown on the map when (i) there are hits to both a chromosome and chromosome Unknown, and (ii) when there are two hits on the same chromosome.

Loci which are underlined displayed homology to a named chromosome and an additional hit to the "unknown" chromosome. The hit to the unknown chromosome may be artefact generated during the assembly process i.e. due to sequence being moved from the unknown chromosome to a named chromosome but not deleted from the unknown chromosome. These include: Aar68, Aar70, Aar84, Aar85, Aar86, Aar87, Aar95, Aar104, Aar113, Aar129, Aar146, Aar152, Aar160, Aar165 and Aar171.

Loci which are displayed in bold and in italics have an additional second hit to the same chromosome.

Those loci could not be mapped to any location using the criteria stated are Aar16, Aar17, Aar20, Aar23, Aar25, Aar28, Aar32, Aar35, Aar48, Aar52, Aar53, Aar55, Aar61, Aar78, Aar80, Aar82, Aar92, Aar102, Aar114, Aar118, Aar131, Aar134, Aar136, Aar137, Aar147, Aar149, Aar153, Aar154, Aar169, Aar175, Aar181, Aar185 and Aar188.
